# Home-Based Telepsychiatry in US Urban Area

**DOI:** 10.1155/2017/6296423

**Published:** 2017-05-29

**Authors:** Alireza Amirsadri, Jaclynne Burns, Albert Pizzuti, Cynthia L. Arfken

**Affiliations:** Department of Psychiatry and Behavioral Neurosciences, Wayne State University, 3901 Chrysler Service Drive, Detroit, MI 48201, USA

## Abstract

Telepsychiatry expands access to psychiatric care. However, telepsychiatry for elderly adults is only reimbursed in the US if the patient is assessed while in a clinical setting. This case study presents a homebound older woman previously hospitalized for schizophrenia who had not seen a psychiatrist in over 20 years. Care was provided with hybrid telepsychiatry (team-based practice with social worker traveling to the home with electronic tablet for connection with psychiatrist). The intervention resulted in detecting unrecognized depression and complex trauma. The treatment plan included adding an antidepressant and therapy plan, eliminating one psychiatric medication, and reducing dosage of pain medication. The outcomes were improved function and quality of life. The patient and caregiver were both highly satisfied with the services. This hybrid telepsychiatry is a reasonable option for homebound elderly patients living in urban areas and less expensive than nursing home admission.

## 1. Introduction

Obtaining quality psychiatric care is challenging in many parts of the world due to shortage of psychiatrists. In the US, there are extensive areas with limited psychiatric coverage [1]. To increase access to physicians in rural areas, the US government passed a law to permit telemedicine to be reimbursed by Medicare (health insurance for adults aged 65 or older, or disabled adults) if the patient is in a clinical facility located in a county outside of a metropolitan area. As of this date, the clinical sites authorized by Medicare are offices of physicians or practitioners, hospitals, critical access hospitals, rural health clinics, Federally Qualified Health Centers, renal dialysis centers affiliated with hospitals or critical access hospitals, skilled nursing facilities, and community mental health centers [2]. Patients' homes are not clinical sites, and Medicare will not reimburse for telemedicine services originating from homes. Only a few entities participating in Federal demonstration projects are exempt from this rule [3] as are private insurance carriers offering supplemental coverage to Medicare-covered patients. This law, while opening access for some patients, does not address challenges in providing psychiatric care in urban areas or to homebound patients. In this case report, we present a hybrid or team-based telepsychiatry intervention with a homebound elderly woman living in an urban area.

## 2. Case Presentation

### 2.1. Presenting Concerns

The patient was a white woman in her mid-80s previously hospitalized for schizophrenia. She lived at home and received care from her only child, a middle age daughter who lived next door. She had a history of psychiatric inpatient hospitalizations with the most recent one in early 1980s. The last time she saw a psychiatrist was “at least 20 years ago” according to the daughter. In the interim, the patient had a series of visiting primary care physicians who cared for her physical health problems and renewed her psychiatric medication prescriptions. At the last visit, the physician informed the patient and her daughter that he was unable to renew the prescriptions as it was not within his or his organization's scope of service. No referral was made to a psychiatrist.

To obtain psychiatric care, the daughter called her insurance carrier who provided some names of psychiatrists. According to the daughter, she called several but none agreed to provide service in the home. After repeated calls to our service, we agreed to provide telepsychiatry in the home.

### 2.2. Findings from Initial Visit

For the home visit, the social worker, accompanied by a peer recovery specialist for safety, carried a 10-inch electronic tablet with secure internet capability. Using a commercially available cloud-based program meeting US privacy standards, the social worker summarized the concerns and history for the psychiatrist who then asked clarifying questions through the social worker or directly to the patient or her daughter.

At the initial visit, the patient was withdrawn with little affect. Despite sitting on the bed at the beginning of the evaluation, she soon laid down and did not respond to questions. The daughter filled in her history including electroconvulsive treatment (ECT). Her mother's hospitalization in early 1980s was prompted after she was found wandering nude in the street. The daughter denied her mother had experienced auditory or visual hallucinations during the past few years. The daughter also stated that the mother had taken stelazine and thorazine in the past but no other psychiatric medications. Currently, she was taking thiothixene 20 mg three times daily (daughter had reduced it to two times daily to avoid running out) and benztropine 1 mg daily. The first visit was brief to limit the strain on the patient.

From the primary care physician, we learned that the patient had been diagnosed with congestive heart failure, degenerative joint disease, type II diabetes, hypothyroidism, and poor vision and hearing. Other medications the patient was taking were glipizide 10 mg daily, lisinopril 10 mg daily, atorvastatin 20 mg daily, metformin 1000 mg twice daily, levothyroxine 75 mcg daily, fish oil 1200 mg 1-2 times daily, ibandronate sodium 150 mg monthly, and tramadol 50 mg three times daily. The primary care physician did not provide additional information. No records were available for any of her psychiatric treatments, either outpatient or inpatient.

Socially, the patient lived alone in a high crime area of a major city. Her daughter, disabled due to shoulder injury, was receiving care for arthritis, anxiety, and depression. A small dog served as the patient's companion; another dog guarded the house. The patient's two-room house, although old, was clean.

### 2.3. Intervention

Changes to care were adding duloxetine 30 mg daily, eliminating benztropine as there were no signs of abnormal movements, and suggesting that tramadol use be minimized or eliminated. Duloxetine was selected as the antidepressant for its effect on pain [4]. Benztropine, medically unnecessary, was also discontinued because in elderly patients it can impair cognition, contribute to development of constipation and blurred vision, and aggravate skin and mucosa dryness [5]. The thiothixene was continued due to stable control of psychotic symptoms and lack of side effects.

The social worker checked for environmental fall risks and the quality and adequacy of the caregiving. The daughter refused referral for visiting nurse services.

### 2.4. First Follow-Up

A follow-up visit was scheduled three months later but was postponed one month as the daughter was unavailable. At this visit, the patient appeared clean and attentive to our questions and interacted appropriately with the dog. She did not know the date but answered other questions quickly and confidently with a smile when she heard them. Her daughter repeated and rephrased some questions so that the patient could understand. She was dressed in a housecoat with warm socks and sat on her bed throughout the visit.

She and her daughter expanded on her history. She was born in rural state, left school after 7th grade, and married soon after. Both the patient and her husband came from large families. Shortly after the marriage, the couple moved approximately 500 miles to the current city where her husband worked as a skilled tradesman. He died six months ago. There was little mention of the marriage but after direct questioning, the daughter admitted that her father slapped her mother after drinking. The patient denied a history of head injuries or surgery. Both the patient and the daughter said there was no history of self-harm or harm to others. Likewise, there was no history of legal involvement. Her drinking history was limited to sips at social events. However, the patient said she smoked tobacco like “a freight train.”

She was hospitalized for psychiatric problems at least three times with the last one in early 1980s lasting for seven days. When the daughter was 7 years old, the patient received 16 rounds of ECT. The daughter particularly remembered that treatment as her mother temporarily forgot how to cook meatloaf when she returned home.

The patient reported that she spent her time sleeping and playing with the small dog. She listened to tapes of Bible reading and watched television. She declared her favorite meal was breakfast and her favorite food was grits. Her only physical activity was using the pot that is located near the bed for toileting. Her weight appeared normal for height. The tramadol use was reduced following the psychiatrist's recommendation to half a 50 mg tablet twice daily from 50 mg three times daily.

### 2.5. Second Follow-Up Visit

At this visit, the patient and the daughter were more relaxed and revealed more information. The daughter switched health insurance plans within the same private carrier to lower out-of-pocket expenses. The new plan did not cover the primary care physician practice they had been using. The daughter was in the process of looking for a new visiting primary care physician. The previous physician had only seen the patient for approximately six months. The daughter also had outside help with caregiving responsibilities.

However, it was revealed that the patient's husband was demanding, yelled, and “got drunk every day,” according to the patient and confirmed by the daughter. Additionally, he pulled the patient around the house by her hair. According to the daughter, her father was anxious, depressed, and paranoid (“everyone was out to get him”). He refused to see a psychiatrist although his primary care physician made a referral. The patient was “relieved” (according to the daughter) that he was gone. For the first time in the daughter's memory, the patient was receiving attention. According to the patient, she was “happy.”

### 2.6. Patient and Caregiver's Perspective

Both the patient and her daughter gave written consent for this case report. They were very happy with the service and wanted it available for others. The daughter said it was “very convenient” and “good care.” The patient declared that she was doing “very well.” For both of them, the telemedicine service was similar to the home visits with the primary care physician. The worst part according to the daughter (who laughed when questioned) was “straightening up the house,” which she also did for the primary care home visits. Both the patient and her daughter were welcoming and said the social worker was the dog's new “girlfriend.”

### 2.7. Outcomes

The hybrid telepsychiatry resulted in detecting undiagnosed depression, discovery of trauma history, and development of therapy treatment plan. In addition, there was reduction of pain medication and improvement in depression. The patient reported improved function and quality of life.

## 3. Discussion

Advantages of this hybrid model of home visit with telemedicine communication with the psychiatrist are access to care for patients who are homebound, no travel time for psychiatrist, and inspection of the home environment by the social worker. Without this collaborative telepsychiatry, the patient's other option to access psychiatric care was to enter a skilled nursing facility although that expensive option would mean leaving her home, daughter, and pet. In 2016, the median cost of a semiprivate room (i.e., sharing a room) in skilled nursing facility in our state was $7,604 per month [6]. Another possible solution for accessing care would involve having a social service agency provide transportation to and from the mental health clinic. However, this would involve considerable coordination as well as physically lifting and bundling her into and out of the vehicle. It also assumes that the patient would be agreeable to the service.

Other advantages of the hybrid model include the social worker being able to assess the home for fall risk and caregiving arrangement and making social service referrals as needed. The nurse, present at the second visit, was able to perform physical examination to obtain blood pressure, pulse, respiration, and temperature as this information was not available from the primary care physician. The nurse also checked for pedal edema, congestion in the lungs, limb strength, coordination, and blood glucose levels. The psychiatrist was able to interact with the patient without leaving the clinic and between clinic visits with other patients. For telepsychiatry, the psychiatrist uses a desktop monitor with webcam at work and 10-inch electronic tablet or mobile phone when at other locations.

Disadvantages of hybrid telepsychiatry are coordinating the visit with the daughter and travel time for the social workers. In rural areas, the travel time might be prohibitive. For this case, the distance between the clinic and patient's home was 10.5 kilometers. Additional challenges in this case were disruption in primary care continuity, insurance coverage changes, and lack of communication from other health providers. Initially there were concerns that the patient would not accept telepsychiatry as it involved interacting with the psychiatrist on an electronic tablet. However, the patient was very attentive and directly answered most questions posed by the psychiatrist. Both the social worker and daughter assisted on rewording some questions which is an advantage of the hybrid model.

Overall, hybrid telepsychiatry is a reasonable option for providing care to urban elderly with transportation and physical challenges.

## References

[1] Hoge M. A., Stuart G. W., Morris J., Flaherty M. T., Paris M., Goplerud E. (2013). Mental health and addiction workforce development: federal leadership is needed to address the growing crisis. *Health Affairs*.

[2] U.S. Department of Health and Human Services, Centers for Medicare and Medicaid Services. Telehealth Services. https://www.cms.gov/Outreach-and-Education/Medicare-Learning-Network-MLN/MLNProducts/downloads/TelehealthSrvcsfctsht.pdf.

[3] Horton K., Malcarney M.-B., Seiler N. (2014). Law and the public's health: medicare payment rules and telemedicine. *Public Health Reports*.

[4] Wang Z. Y., Shi S. Y., Li S. J. (2015). Efficacy and safety of duloxetine on osteoarthritis knee pain: a meta-analysis of randomized controlled trials. *Pain Medicine*.

[5] Mintzer J., Burns A. (2000). Anticholinergic side-effects of drugs in elderly people. *Journal of the Royal Society of Medicine*.

[6] Genworth Financial Inc.. https://www.genworth.com/about-us/industry-expertise/cost-of-care.html.

